# Study of Lignin-Modified Silica Gel Adsorption after Association with Six Different Organophenylmercuric Compounds in Chloroform

**DOI:** 10.3390/ijms19102851

**Published:** 2018-09-20

**Authors:** Shushi Chen, Yu-Xuan Gao

**Affiliations:** Department of Applied Chemistry, National Chiayi University, Chiayi 600, Taiwan; s1050360@mail.ncyu.edu.tw

**Keywords:** lignin-modified silica gel, π-π complexation, organophenylmercuric compound, theoretical interaction simulation, dipole-dipole interaction

## Abstract

In this study, the adsorption of lignin-modified silica gel after association with six different organophenylmercuric compounds in chloroform was investigated. Adsorption reached approximately 90% of the maximum value within 15 min. The adsorption capacity, Fourier transform infrared spectroscopy, and interaction simulation results indicated that the adsorption proportion resulted from the strong dipole-dipole interaction between the lignin and analyte molecules, and was considered to be size- and structure-dependent. However, the π-π complexation interaction arising from the acidic aromatic moiety of the analyte, which was significant in an apolar environment, was not the major force responsible for the resulting adsorption. Additives, such as acid or ether, which competed with the analyte for the binding site on the lignin molecule, were not beneficial to the interaction, and thus not beneficial to the adsorption processes.

## 1. Introduction

Consumption of marine fish and shellfish contaminated by organomercuric compounds, such as methylmercury, has exposed 75% of humans worldwide to mercury [1,2,3]. In the United States, human mercury exposure resulting from consumption of tuna harvested from the Pacific Ocean was estimated to be 40% [4,5]. The organomercury compound is readily formed because of mercury’s strong affinity with the thiol group, further complicating the toxicological properties of the mercury. The cited studies have also addressed how metal mercury emitted from factories and from the burning of coal and waste turns into organomercury. Algae produced near the surface of sunlit waters die quickly and sink. The settling algae are then decomposed by bacteria, and the interaction of this decomposition process with mercury results in the formation of organomercury, which is then spread through the ecological system. Alkylmercurys, such as methylmercury and ethylmercury, are typical compounds produced in this process.

Many capillary electrophoresis (CE)- [6,7], gas chromatography (GC)-, and high-performance liquid chromatography (HPLC)-related [8,9,10,11,12,13,14,15,16] separation techniques associated with preconcentration extraction methods had, in some cases, been developed prior to the analysis of inorganic mercury or oganomercury present in the environment or in biological samples. The extraction mechanism leading to the analyte’s preconcentration was involved in either the complexation or exchanging of mercury species with a sulfur-containing agent. An extraction efficiency higher than 95.0% has been reported. On the other hand, numerous direct spectroscopic approaches for detecting these mercury species in aqueous solution have been reported [17,18]. However, the removal of organophenylmercury species from polluted sites has rarely been reported, except for phenylmercuric chloride. In the present study, lignin, immobilized on the surface of silica gel without further treatment, was used as an adsorbent for six different organophenylmercuric compounds in chloroform. Adsorption capacity data were acquired, and Fourier transform infrared spectroscopy (FTIR) and a theoretical interaction simulation were performed to explore the mechanism involving the adsorption process. The effects of using additives in the liquid phase were also studied.

## 2. Results and Discussion

In this study, the following organophenylmercuric compounds were examined: phenylmercuric chloride, phenylmercuric acetate, 4-aminophenylmercuric acetate, phenylmercuric salicylate, thiomersal, and phenylmercuric dimethyldithiocarbamate. As the nomenclature indicates, all of these analytes, except thiomersal, share the same cationic phenylmercuric moiety. The only structural differences between the analytes are their anionic moieties. These anionic moieties can be structurally classified into the categories, ester and thioester.

### 2.1. Mechanism Leading to Adsorption

**Figure 1:**
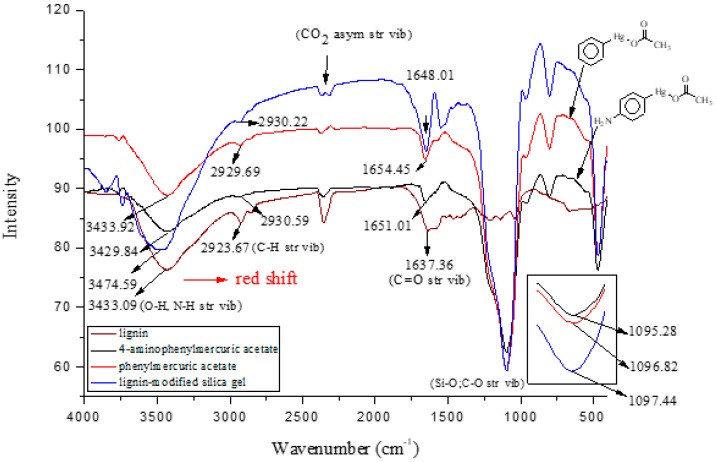
The superimposed Fourier-transform infrared spectroscopy (FTIR) spectra for phenylmercuric acetate, 4-aminophenylmercuric acetate, lignin and lignin-modified silica gel for easy, quick reference and comparison.

**Figure 2:**
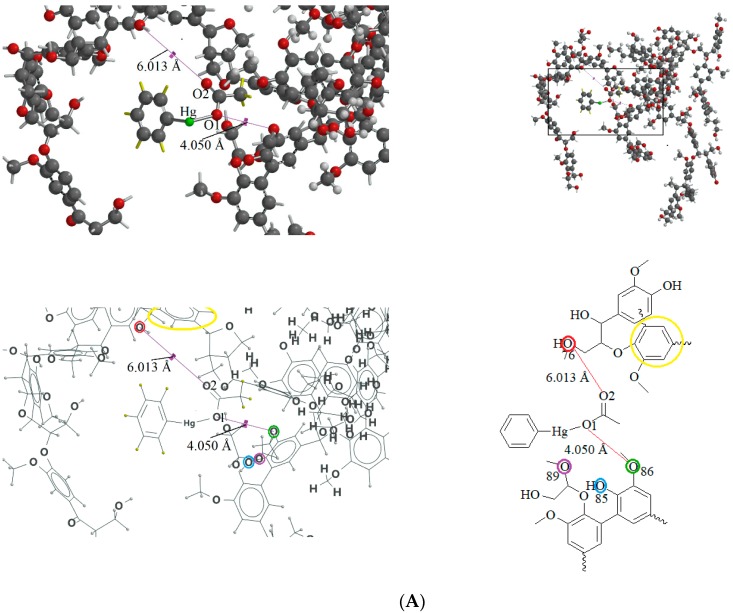

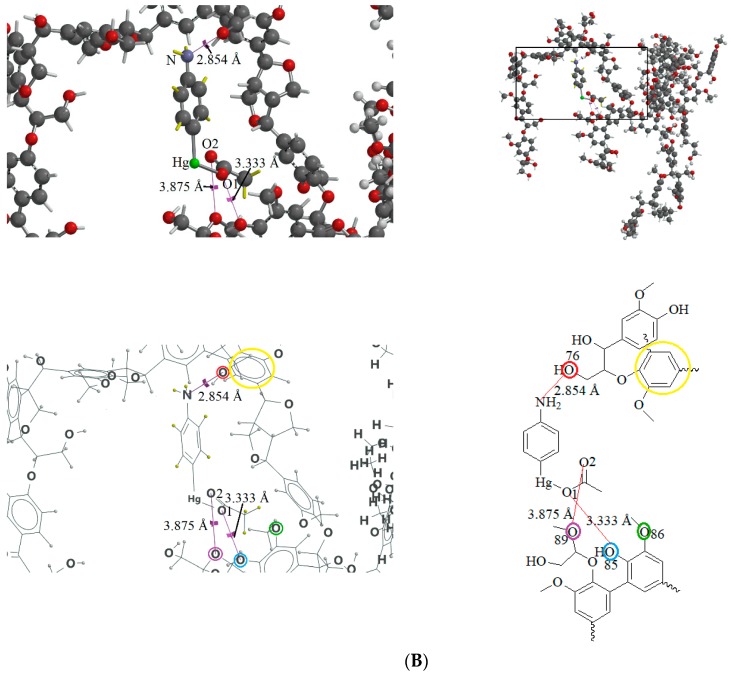
The theoretical interaction simulation between lignin and phenylmercuric acetate (**A**), and 4-aminophenylmercuric acetate (**B**) molecules, respectively under energy-minimized conditions. Oxygen atoms are in red in the ball-and-stick model. The oxygen atoms circled in red, green, and blue are the same oxygen atom in both the 3D and 2D molecular structure drawings for keeping track, as well as for the perception of mutual orientation alteration of molecules in the two cases. An aromatic ring is also circled in yellow for the same purpose. Note that the hydrogen atom of analyte is in yellow in the 3D structure drawing for distinction purposes. Only the crucial part of the structure is shown in the 2D drawing for simplicity.

Chloroform is an apolar solvent, and its dielectric constant and the corresponding autoprotolysis constant are relatively small compared with those of water (4.8 vs. 80). Consequently, in a chloroform matrix, substances that are electrolytic in nature or are ionizable are not separated into free, positively and negatively charged species—rather, they exist in intimate ion pairs [19]. The interaction of the organophenylmercuric analytes with the lignin-modified silica gel was thus considered to be on a molecular level in chloroform matrix, as opposed to the level of cationic or anionic moieties of the analytes. This conclusion was fully supported by the adsorption data for these analytes (summarized in Table 1). The adsorption proportions varied under the same evaluation conditions. If the interaction had not occurred on the molecular level, the adsorption for all examined analytes would either have remained constant or have been unobservable, depending on which ionic moiety was involved in the interaction with the lignin-modified adsorbent molecule. Furthermore, the adsorption capacities determined for these analytes would have been different from those listed in Table 1. The adsorption capacities were determined to be larger than those previously reported for organophosphate pesticides on humic-fraction-modified silica gel in hexane, and were of approximately the same order of magnitude as that reported for the adsorption of triazines on a lignin-modified adsorbent in hexane [20]. In the cited studies, the adsorption was determined to have resulted from intense dipole-dipole interactions (e.g., hydrogen bonding) between the analytes and the adsorbent molecules in hexane. In the present study, this type of interaction was observed in FTIR results for the adsorption of phenylmercuric analytes in chloroform, particularly for phenylmercuric acetate and 4-aminophenylmercuric acetate in Figure 1, with other spectra of lignin and lignin-modified silica gel superimposed for easy and rapid reference and comparison. The analytes’ amino groups at the para position represent their only difference in structure. Close examination revealed a red shift in the region centered at 3474.59 cm^−1^ for O–H and N–H stretching vibrations. The shift was considered significant in magnitude, and was attributed to the interaction between the functional groups of hydroxyl and carboxyl on the adsorbent and analyte molecules, respectively. More shifts in frequency were recorded when the examined analyte was aminated at the para position, indicating additional involvement of the amino group in the dipole-dipole interaction. This conclusion was supported by the theoretical interaction simulation presented in Figure 2. In the simulation, the energy for the analyte and the adsorbent (structure partially displayed) was minimized, and calculations were then continuously performed until the minimum energy for the mutual orientation of the two was reached. Subsequently, the shortest distance between selected oxygen-containing functional groups in the orientation was determined. As illustrated in the figure, the mutual orientation of the adsorbent and phenylmercuric acetate was altered when the analyte was replaced with its aminated version. For example, the shortest distance between the oxygen atom of the carbonyl group, designated as O2 on the analyte, and the oxygen atom of the hydroxyl group, marked by the red circle for keeping track, on the lignin was 6.013 Å in the case of phenylmercuric acetate. However, an additional dipole–dipole interaction was introduced as the analyte was aminated, which shortened the distance to 3.875 Å. The oxygen (in the purple circle in the figure) on the lignin was then part of the ether linkage. Critically, the overall energy released upon association was further minimized to −12158.898 from −12148.771 (kJ/mol) because of the additional dipole-dipole interaction of the amino group and the shorter O–O distance, which enhanced the opportunity for interactions with other atoms surrounding the indicated oxygen atom. In other words, the orientation of the two molecules became more stable upon their association due to the presence of the amino group. This phenomenon may also explain why the adsorption proportion for 4-aminophenylmercuric acetate was approximately 100% but was only 72.84% for aminophenylmercuric acetate under the same adsorption evaluation conditions (Table 1).

### 2.2. Effect of An Additive in the Liquid Phase on Adsorption

**Figure 3:**
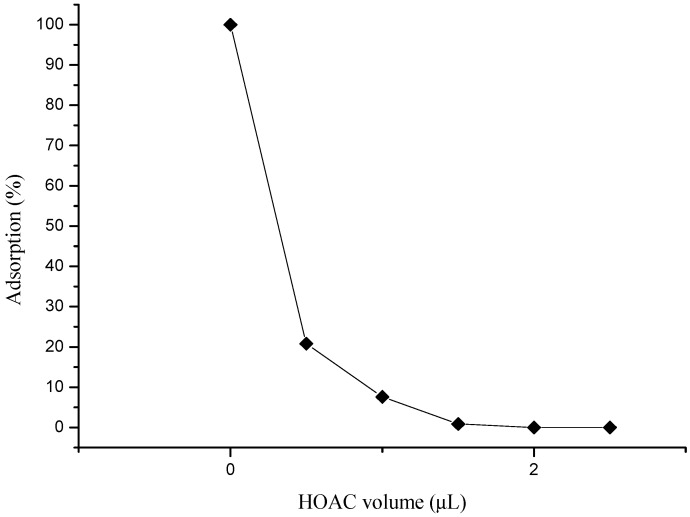
The effect of acidic additive on the percentage of adsorption of 4-aminophenylmercuric acetate on lignin-modified silica gel.

To further investigate the adsorption mechanism of the dipole-dipole-based interaction, ether and acid additives were separately introduced into the liquid phase. The proportion of adsorption of 4-aminophenylmercuric acetate on lignin-modified silica gel, presented in Figure 3, decreased considerably upon the addition of acetic acid in the chloroform. Deteriorated adsorption was also observed in the case with an ether additive (not shown). This result was attributed to the interaction competition between the analyte and the additive molecules for the available binding sites on the adsorbent molecule. The results also indicated that the protonation of the analyte in the acidic liquid phase was not beneficial to the adsorption process.

### 2.3. Effects of Functional Group and Size of Analyte on Adsorption

**Figure 4:**
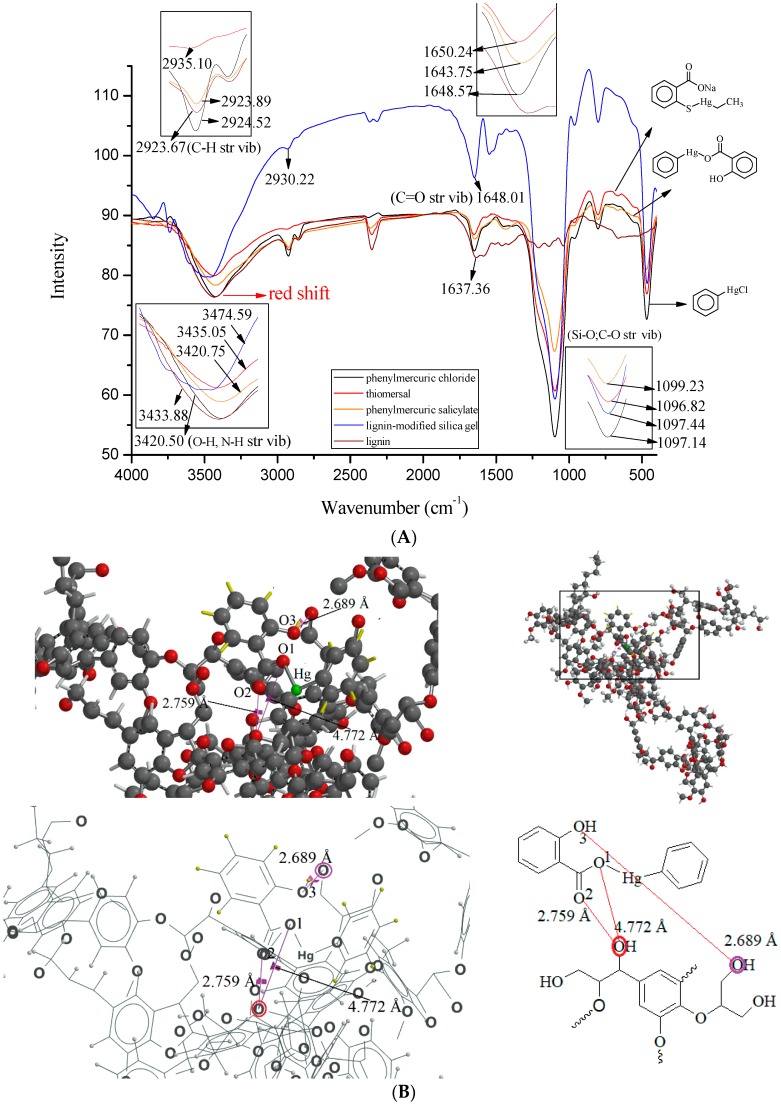
The superimposed FTIR spectra of phenylmercuric salicylate, thiomersal, phenyl mercuric chloride, lignin and lignin-modified silica gel for easy, quick reference and comparison (**A**), and the theoretical interaction simulation between lignin and phenylmercuric salicylate (**B**) after the energy minimization. Note that the hydrogen atom of the analyte in the 3D structure drawing is in yellow for easier distinction. The colored oxygen atoms, interacting with the carboxylate and hydroxyl groups of analyte on the lignin molecule, can be better viewed on a 2D structure drawing on the right.

**Figure 5:**
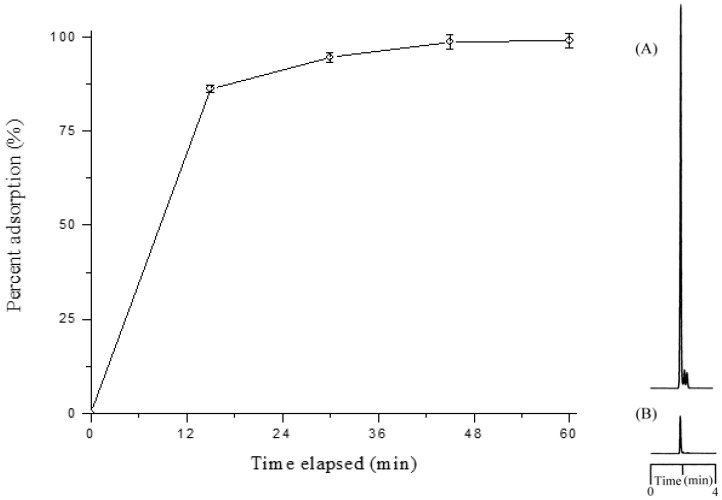
The relationship between the percentage of adsorption for phenylmercuric salicylate and the contact time during a 1-h process. The chromatograms show the standard (**A**), and the percentage of adsorption for 15 min contact time (**B**). The mobile phase of 491/10/1/1 by volume (acetonitrile/methanol/acetic acid/triethylamine, *v*/*v*) was used in the evaluation. The UV detection wavelength was set at 259 nm.

The adsorption proportion and capacity data (summarized in Table 1) also indicated that analytes with higher numbers of functional groups for interaction with the lignin molecule corresponded with higher proportions of adsorption. Examples of such analytes include phenylmercuric salicylate and phenylmercuric dimethyldithiocarbamate. As indicated in Figure 4A, the FTIR study of phenylmercuric salicylate revealed a significant red shift in O–H as well as N–H, stretching vibrations caused by an intense dipole-dipole interaction. These results were attributed to the hydroxyl group on the aromatic moiety because the magnitude of the red shift was comparable to those observed in the cases of phenylmercuric acetate and 4-aminophenylmercuric acetate. The interaction simulation results presented in Figure 4B indicate that the O–O distance became shorter due to the additional dipole-dipole interaction between the hydroxyl group and the aromatic moiety. The two molecules moved closer to each other and released additional energy (−12204.997 kJ/mol) to form a more stable conformation. The analyte appeared to have been embedded in the cleft of the lignin molecule. As expected, the proportion of adsorption for phenylmercuric salicylate for a 1 h contact time in Figure 5 (left) reached approximately 100% under the evaluation conditions. Typical chromatograms in Figure 5 (right) revealed adsorption of 86.21% in 15 min, indicating that the adsorption process was still rapid in chloroform, which is more viscous than hexane.

**Figure 6:**
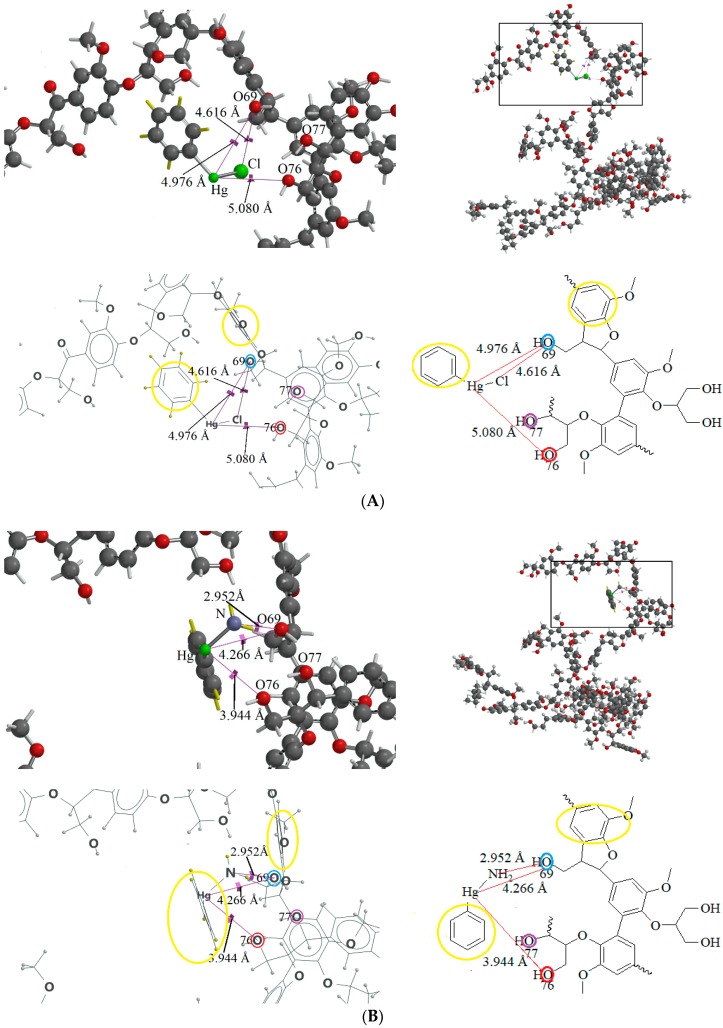
The theoretical interaction simulations between lignin and phenylmercuric chloride (**A**) and phenylmercuric amine (**B**) after the energy minimization. The same atoms in both 3D and 2D molecular structure drawings are colored for tracking purposes, as well as for the perception of mutual orientation alteration of molecules in the two cases. Aromatic rings are also circled in yellow for the same purpose. Note that the hydrogen atom of analyte in the 3D structure drawing is in yellow for easier distinction. Only the crucial part of the structure is shown in the 2D drawing for simplicity and clarity.

Phenylmercuric chloride is unique among the analytes examined in this study because its structure does not involve oxygen- or sulfur-containing moieties. However, an FTIR red shift, comparable to that for phenylmercuric salicylate in the region of O–H and N–H stretching vibrations, was also observed in phenylmercuric chloride (Figure 4A). These results were attributed to the dipole-dipole interaction involving the electronegative chlorine atom. The interaction simulation represented in Figure 6A indicated that the shortest Cl–O69 distance was 4.616 Å. The amount of energy released corresponding to this stable conformation was −11,942.019 kJ/mol, which was much less than that for phenylmercuric salicylate (−12,204.997 kJ/mol). The hindrance effect from the bulky chlorine atom of the analyte during movement toward the lignin molecule appeared to be responsible for the relatively small amount of energy released, which in turn resulted in a relatively low proportion of adsorption under the same evaluation conditions (Table 1). The hindrance effect was further studied in the same region of the lignin molecule by replacing a chlorine atom with an amino group in the interaction simulation, as depicted in Figure 6B, and a much smaller N–O69 distance was measured (2.952 Å). Nitrogen and chlorine atoms are all electronegative, and thus capable of dipole-dipole interactions. However, the nitrogen atom creates less of a steric hindrance effect because it is smaller than the chlorine atom.

## 3. Material and Methods

### 3.1. Apparatus

The HPLC system used in this study was a Hitachi Model L-7100 attached to a D-2500 Chromatopac data station and an ultraviolet (UV) detector. The detection wavelength used for adsorption and capacity evaluations was set at 259 nm in all cases. FTIR spectra with a resolution of 4 cm^−1^ for analytes pelleted in potassium bromide (KBr) were acquired by scanning samples 10 times on a Shimadzu Model FTIR-8400 system. In all cases, a 15-cm C_18_ column was used.

### 3.2. Chemicals

The organosilane reagent used as a linker in chemical immobilization reactions and the organomercuric compounds studied in this study were purchased from Sigma (St. Louis, MO, USA) and the Aldrich Chemical Co. (Milwaukee, WI, USA), respectively. Silica gel (5-µm particle diameter, 100 Å porosity with a specific surface area of 400 m^2^/g), used as the supporting matrix of the solid phase and as the adsorbent in the adsorption and capacity evaluations at ambient temperatures after chemical modification with a lignin without further purification, was obtained from Silicycle (Quebec City, QC, Canada) [21,22]. The HPLC-grade solvents, including toluene, acetonitrile, methanol, triethylamine, methylene chloride, hexane, chloroform, and ethyl ether used in this study were purchased from Fisher Scientific (Pittsburgh, PA, USA). These solvents were used to wash the lignin-modified silica gel before evaluations of adsorption and adsorption capacity, as well as for the mobile phase in HPLC analysis. Filtered (0.2 µm) and distilled water was used in all cases.

### 3.3. Conditions for Measuring the Proportion of Adsorption and the Adsorption Capacity

**Figure 7:**
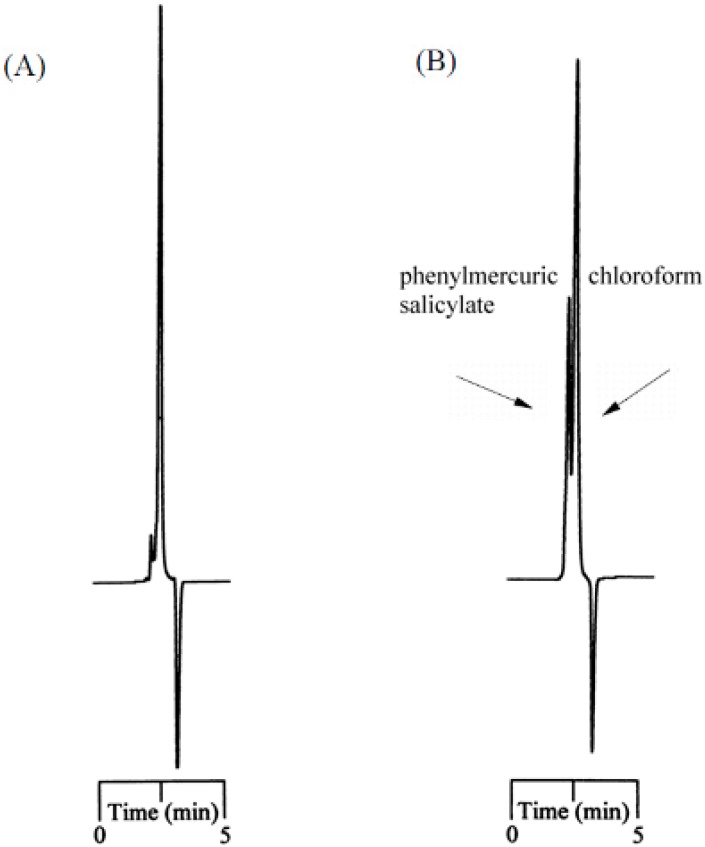
Chromatograms for the elution of phenymercuric salicyalte after (**A**) and before (**B**) adsorption on the lignin-modified silica gel in chloroform using the mobile phase of 491/10/1/2 by volume (acetonitrile/methanol/acetic acid/triethylamine, *v/v*). The UV detection wavelength was set at 259 nm.

100-μL of a 1.57 × 10^−3^ M standard solution of a selected analyte and 10 mg of a lignin-modified adsorbent were mixed for a controlled period of time. The solution was sampled for HPLC analysis both before and after the adsorption process in each measurement, and the proportion of adsorption was then calculated according to the differences in peak areas. A standard solution was added to the matrix in 25–100-μL increments until a detectable UV signal was recorded. This signal was then used to determine the adsorption capacity. The HPLC measurement was in triplicate to obtain a mean value. To avoid interference from the chloroform as a liquid phase in adsorption—illustrated in Figure 7 for analytes such as phenylmercuric salicylate at a UV detection wavelength of 259 nm—the evaluations were performed in acetonitrile after evaporation.

### 3.4. Theoretical Computations Performed with Spartan 14 Software

A semiempirical molecular orbital calculation method for single-point energy was conducted (Parameterized Model 3) with Spartan 14 software (Wavefunction, Irvine, CA, USA). Prior to theoretical calculation, the molecular energy was first minimized by continuously altering the bond angle and orientation until a conformation with minimum energy was identified. Interactions of atoms on both the lignin and analyte molecules were then theoretically simulated and calculated until the lowest formation energy at the ground state was determined (i.e., the heats of formation) [23]. Herein, only the portion of the lignin structure corresponding to the lowest formation energy in the interaction simulation is displayed for lucid local magnification.

## 4. Conclusions

Molecular-level adsorption of lignin-modified silica gel after association with six different organophenylmercuric compounds was achieved within 15 min in chloroform. The adsorption, based on the measured adsorption capacities, the FTIR, and the interaction simulation results of the adsorption reactions were attributed to the strong dipole–dipole interactions between the lignin, electronegative nitrogen, sulfur, or oxygen atom-containing moiety of analyte molecules. The π-π complexation interaction involving an aromatic moiety, significant in an apolar environment, was not the major force responsible for the adsorption. Due to the resultant competition with analytes for the binding site on the lignin molecule, additives of acid and ether were not beneficial to the adsorption interaction, and thus detrimental to the adsorption process.

## Figures and Tables

**Table 1:** The percentage of adsorption and selected adsorption capacity for six different phenylmercuric compounds with lignin-immobilized silica gel as the adsorbent in chloroform ^a^.

Compound	Structure	Adsorption Capacity (%) ^b^	Adsorption (%) ^b^
4-Aminophenylmercuric acetate	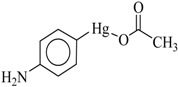	0.301 ± 0.031	~100
Phenylmercuric acetate	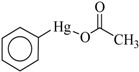	-	72.84
Phenylmercuric chloride	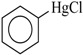	-	50.36
Phenylmercuric dimethyldithiocarbamate	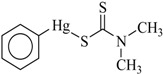	0.281 ± 0.021	~100
Phenylmercuric salicylate	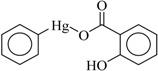	0.312 ± 0.019	~100
Thiomersal	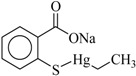	0.279 ± 0.018	~100

^a^ The reported percentage of adsorptions was measured over a time period of 1 h in chloroform. The amount of lignin-modified silica gel used as the solid phase in each triplet adsorption measurement was 10 mg. The volume of liquid phase containing analyte was 100 µL. The percentage of adsorption (%), an average of three measurements, was calculated based on the difference in peak area of the analyte before and after the adsorption. ^b^ The mobile phase used in the adsorption capacity evaluation was a HPLC-grade solvent mixture of acetonitrile/methanol/acetic acid/triethylamine by volume (490/10/1/1, *v/v*) for all analytes. The UV detection wavelength was at 259 nm.
